# The *AINTEGUMENTA* genes, *MdANT1* and *MdANT2*, are associated with the regulation of cell production during fruit growth in apple (*Malus* × *domestica* Borkh.)

**DOI:** 10.1186/1471-2229-12-98

**Published:** 2012-06-25

**Authors:** Madhumita Dash, Anish Malladi

**Affiliations:** 1 Department of Horticulture, 1111 Miller Plant Sciences, University of Georgia, Athens, GA, 30602, USA

**Keywords:** Cell division, Cell expansion, Fruit development, Fruit size, Organ growth

## Abstract

**Background:**

Fruit growth in apple (*Malus* × *domestica* Borkh.) is mediated by cell production and expansion. Genes involved in regulating these processes and thereby fruit growth, are not well characterized. We hypothesized that the apple homolog(s) of *AINTEGUMENTA* (*ANT*), an APETALA2–repeat containing transcription factor, regulates cell production during fruit growth in apple.

**Results:**

Two *ANT* genes, *MdANT1* and *MdANT2*, were isolated from apple and their expression was studied during multiple stages of fruit development. *MdANT1* and *MdANT2* expression was high during early fruit growth coincident with the period of cell production, rapidly declined during exit from cell production, and remained low during the rest of fruit development. The effects of increase in carbohydrate availability during fruit growth were characterized. Increase in carbohydrate availability enhanced fruit growth largely through an increase in cell production. Expression of *MdANT1* and *MdANT2* increased sharply by up to around 5-fold in response to an increase in carbohydrate availability. Expression of the *ANT* genes was compared across two apple genotypes, ‘Gala’ and ‘Golden Delicious Smoothee’ (GS), which differ in the extent of fruit growth, largely due to differences in cell production. In comparison to ‘Gala’, the larger fruit-size genotype, GS, displayed higher levels and a longer duration of *MdANT1* and *MdANT2* expression. Expression of the *ANTs* and cell cycle genes in the fruit core and cortex tissues isolated using laser capture microdissection was studied. During early fruit growth, expression of the *MdANTs* was higher within the cortex, the tissue that constitutes the majority of the fruit. Additionally, *MdANT1* and *MdANT2* expression was positively correlated with that of A- and B-type *CYCLINS*, B-type *CYCLIN-DEPENDENT-KINASES* (*CDKBs*) and *MdDEL1*.

**Conclusions:**

Multiple lines of evidence from this study suggest that *MdANT1* and *MdANT2* regulate cell production during fruit growth in apple. ANTs may coordinate the expression of cell proliferation genes and thereby affect the competence of cells for cell production during fruit growth. Together, data from this study implicate *MdANT1* and *MdANT2* in the regulation of fruit growth in apple.

## Background

Apple (*Malus* × *domestica* Borkh.) fruit growth is mediated by cell production and expansion. After bud-break, rapid growth within the ovary and floral-tube tissues is facilitated by intensive cell production. This phase is followed by a period of temporary cessation of growth around bloom associated with quiescence in cell production, a phenomenon which likely prevents fruit growth in the absence of pollination and fertilization [1]. Cell production is re-initiated in response to signals generated during pollination and/or fertilization resulting in fruit set. Early fruit development is associated with intensive cell production-mediated growth which occurs until several weeks after fruit set [1-3]. Final cell number attained by the end of this period contributes greatly to the sink strength and thereby the growth potential of the fruit. Subsequent fruit growth is associated with post-mitotic cell expansion, a process which continues until maturity and contributes to the majority of fruit growth and increase in fruit size [1,3]. Enhanced fruit growth and increase in fruit size are mediated by changes in cell production or expansion. For example, increase in fruit growth under higher carbohydrate availability during early fruit development is primarily associated with an increase in cell production [4]. Also, variation in fruit growth potential and fruit size across genotypes is associated with differences in cell number and size [3,5]. Although it is apparent that cell production and expansion are important determinants of fruit growth, our understanding of the molecular mechanisms and genes that regulate these processes remains limited.

Cell production during fruit growth is potentially regulated by genes controlling the progression of the cell cycle [1,6,7]. Previous research indicated coordinated changes in the expression of core cell cycle genes during different phases of fruit growth in apple [1]. Expression of 14 such genes including B-type *CYCLIN DEPENDENT KINASES* (*CDKs*), A- and B-type cyclins, a WEE kinase (*MdWEE1*), and an atypical E2F transcription factor (*MdDEL1*) was positively associated with cell production during fruit growth and development. These genes displayed high expression before bloom and during early fruit development, stages primarily associated with rapid growth mediated by cell production. Subsequently, these genes displayed a sharp reduction in expression coincident with exit from cell production during fruit development. Additionally, five cell cycle genes including the *KIP RELATED PROTEINS* (*KRPs*), *MdKRP4* and *MdKRP5*, were negatively associated with cell production during different phases of fruit growth and development. It is likely that upstream regulatory genes may, either directly or indirectly, coordinate changes in the expression of these cell cycle genes as well as other genes associated with cell proliferation, thereby regulating cell production during fruit growth. Such upstream regulators of cell production during fruit growth have not yet been definitively identified in apple. Recently, an *AUXIN RESPONSE FACTOR* (*ARF106*) expressed during cell division and expansion phases of apple fruit development was co-localized to a major fruit size QTL, suggesting its involvement in regulating fruit growth [8]. In other fleshy fruit such as tomato (*Solanum lycopersicum*), *FW2.2*, a fruit size regulator, inhibits cell production potentially through its association with a cell cycle gene, and thereby regulates fruit growth [9,10]. Also, *SUN*, a gene involved in the regulation of tomato fruit shape may affect the patterns and orientations of cell proliferation during early fruit growth [11,12]. Beyond the above examples, little information is available regarding upstream regulators of cell production during growth of fleshy fruit. Identification and characterization of such genes is essential to develop a better understanding of fleshy fruit growth.

Genes controlling organ growth are potential candidates for the regulation of growth of fleshy fruit. Many genes that regulate organ growth have been identified in Arabidopsis (*Arabidopsis thaliana*) and other plants [13-15]. One such gene, *AINTEGUMENTA* (*ANT*) is a key regulator of organ growth in Arabidopsis. *ANT* is involved in the regulation of ovule development, floral organ growth and development, and organ size in Arabidopsis [16-21]. Arabidopsis *ant* mutants display pleiotropic effects including a reduction in the size of floral organs and leaves [16,17,19]. Over-expression of *ANT* in Arabidopsis results in an increase in the duration of cell proliferation and enhances organ size in leaves, floral organs and siliques [19]. Additionally, ANT mediates the effects of other genes involved in regulating organ growth. In Arabidopsis, *ARGOS* (*AUXIN-REGULATED GENE INVOLVED IN ORGAN SIZE*) promotes cell production and growth, and positively regulates final organ size in an auxin-dependent manner [22]. Over-expression of *ARGOS* in Arabidopsis increases *ANT* expression, and the effects of *ARGOS* on organ growth are attenuated in the *ant* mutant, suggesting that *ANT* mediates *ARGOS*-dependent effects of auxin on organ growth. *ANT* expression is also affected by *AUXIN RESPONSE FACTOR2* (*ARF2*), a negative regulator of cell production and organ size in Arabidopsis [23].

*ANT* is a member of the APETALA2/ETHYLENE RESPONSE FACTOR (AP2/ERF) family of transcription factors and is grouped within the AP2 sub-family. Members of the AP2 sub-family are defined by the presence of two AP2 domains separated by a conserved linker region which together constitute the DNA binding domain [24,25]. Genes within the AP2 sub-family, including several *AINTEGUMENTA-LIKE* (*AIL*) genes, are involved in the regulation of a multitude of plant growth and developmental processes. For example, *APETALA2* (*AP2*) is involved in determining floral organ identity, regulating flower development, maintaining the stem cell niche in the shoot apical meristem, and regulating seed size [26-29]. *AP2* negatively regulates replum growth and valve margin formation during Arabidopsis fruit development [30]. *PLETHORA* (*PLT*) genes are *AILs* which function as master regulators of root growth and development in Arabidopsis partly through their effects on promoting cell proliferation [31,32]. *AtBBM* (*BABYBOOM/AIL2*) promotes cell production, and regulates embryo development and root growth [32,33]. Additionally, *AIL5* and *AIL6/PLT3* are positive regulators of cell production and organ growth in Arabidopsis as their over-expression leads to enhanced floral organ growth [21,34,35].

Whether *ANT* and/or the *AIL* genes are involved in regulating the growth of fleshy fruit has not been investigated previously. It was hypothesized that the apple *ANT* homolog(s) regulate cell production during fruit development and therefore contribute to regulation of fruit growth. Here, the isolation and characterization of two *ANT* genes from apple is reported. Evidence supporting the role of these genes in regulating cell production during different stages of fruit growth, across genotypes differing in fruit growth potential, and in response to carbohydrate availability is presented. Data from this study implicate *ANTs* in the regulation of fleshy fruit growth.

## Results

### Isolation of the apple *ANT* genes

**Figure 1:**
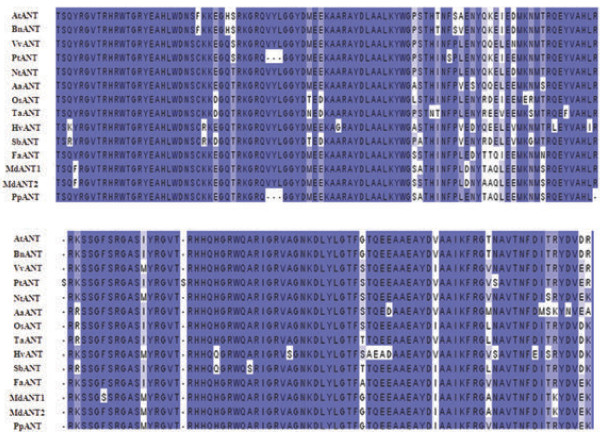
**Comparison of predicted amino acid sequences within the DNA binding domain of ANT from higher plants.** The predicted amino acid sequence of the two AP2 domains and linker region are shown for 14 ANTs from 13 plants. Sequences were aligned using MUSCLE software. The scientific name along with the protein name and the region corresponding to the AP2 domains and the linker regions, is indicated within the parenthesis. *Arabidopsis thaliana* (AtANT; 280-451)*, Brassica napus* (BnANT; 284-454)*, Artemisia annua* (AaANT; 157-327), *Triticum aestivum* (TaANT; 283-453), *Oryza sativa* (OsANT; 289-459), *Hordeum vulgare* (HvANT; 293-463), *Sorghum bicolor* (SbANT; 216-386), *Malus × domestica* (MdANT1; 291-462), *Malus × domestica* (MdANT2; 289-460), *Prunus persica* (PpANT; 305-471), *Fragaria vesca* (FvANT; 260-430), *Nicotiana tabacum* (NtANT; 309-479), *Vitis vinifera* (VvANT; 256-426)*, Populus trichocarpa* (PtANT; 265-434).

Eight expressed sequence tags (ESTs) with homology to the Arabidopsis *ANT* were identified from publicly available apple EST databases*.* The EST displaying the highest similarity with the *AtANT* was designated as *MdANT* and selected for the isolation of the full-length gene. The 3′ RACE analysis of *MdANT* revealed the presence of two *ANT* genes which were designated as *MdANT1* and *MdANT2*. Full-length sequences of these genes were determined as described in the ‘Methods’ section. *MdANT1* and *MdANT2* shared 93% homology at the nucleotide level (coding region) and 90% identity at the amino acid level. Nucleotide sequence identity also extended into the 5′ (~1 kb) and 3′ (~0.5 kb) regions of the open reading frame. Both genes encode putative protein products with 651 amino acids. Phylogenetic analysis of different plant ANT transcription factors, including MdANT1 and MdANT2, using their predicted protein sequences is shown in Additional file 1. MdANT1 and MdANT2 displayed higher sequence similarity with AtANT than with the other AILs from Arabidopsis (Additional file 1). MdANT1 and MdANT2 shared >50% amino acid identity with the Arabidopsis ANT and >75% identity with the peach (*Prunus persica*) ANT. MdANT1 and MdANT2 displayed high sequence similarity with other plant ANTs within a stretch of around 170 amino acids containing the AP2-domain repeats and the linker region (Figure 1). MdANT1 and MdANT2 displayed greater than 88% identity with AtANT in this region. MdANT1 and MdANT2 contained a basic motif (TKKR) similar to the nuclear localization signal in AtANT (KKKR; [36]). Nineteen amino acids within the two AP2-domain repeats and the linker region essential for the DNA binding activity of AtANT were identified in Arabidopsis [25]. All of these residues were conserved within the two apple ANTs. Seven potential apple *AILs* were identified from the EST databases and subsequently five were confirmed following comparisons with the apple genome database (Additional file 1). All these genes contained the well conserved AP2-domain repeats and the linker region.

### Expression of *MdANT1* and *MdANT2* is associated with cell production during fruit growth

**Figure 2:**
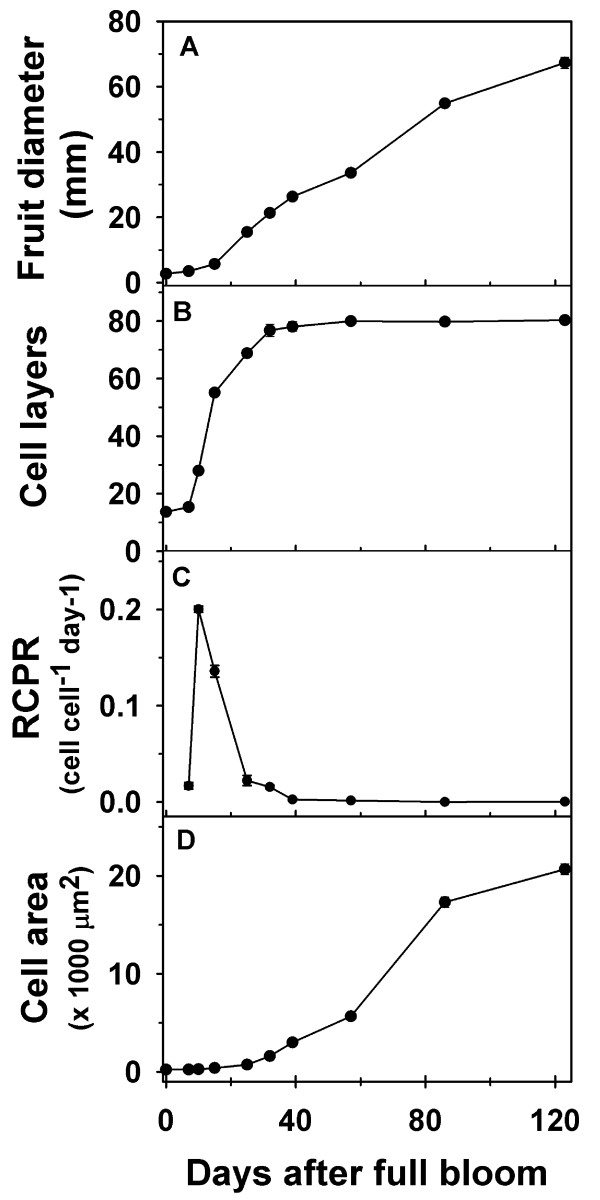
**Fruit and cell growth-related parameters during fruit development in ‘Gala’.** (**A**) Fruit diameter, (**B**) cell number (layers), (**C**) relative cell production rate (RCPR) and (**D**) cell area were measured from full bloom until maturity. Error bars represent standard error of the mean and are smaller than the symbol when not visible. Four biological replicates were used in this study (*n* = 4).

Fruit diameter in ‘Gala’ increased by over 4-fold between 7 and 25 DAFB (days after full bloom) and continued to increase linearly during the rest of fruit development (Figure 2A; Additional file 2). Analysis of cell production within the fruit cortex indicated little change in cell number between 0 DAFB and 7 DAFB (Figure 2B). A rapid increase in cell number (3.6-fold) was observed between 7 DAFB and 15 DAFB. This was also reflected in the high relative cell production rates (RCPR) observed especially around 10 and 15 DAFB (Figure 2C). While the cell number continued to increase between 15 and 32 DAFB (Figure 2B), this occurred at a slower rate than that between 7 and 15 DAFB. The RCPR declined rapidly during this period, and reached basal levels by around 32 DAFB. Cell number did not change greatly after this period. The cortex cell area displayed little change during early fruit growth but increased from around 25 DAFB, coincident with the period of decline in cell production (Figure 2D). Most of the increase in cell area occurred during the later stages of fruit development and was associated with the majority of increase in fruit size (Figure 2D).

**Figure 3:**
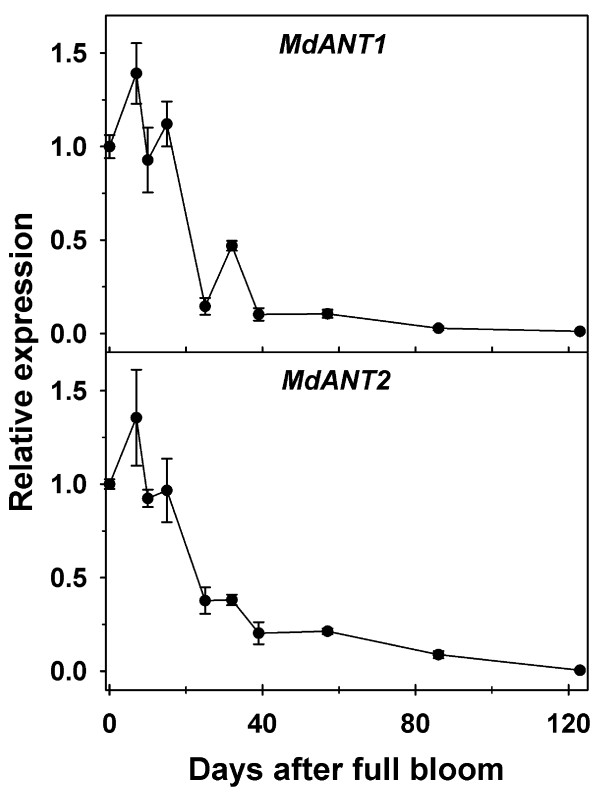
**Expression of*MdANT1*and*MdANT2*during fruit development in ‘Gala’.** Fold-change in the expression of a gene relative to its expression at full bloom (0 DAFB) is presented here. *MdACTIN* and *MdGAPDH* were used as the reference genes. Error bar represents the standard error of the mean of four biological replicates (*n* = 4).

*MdANT1* and *MdANT2* displayed largely similar patterns of expression during fruit development (Figure 3; Additional file 2). Expression of *MdANT1* and *MdANT2* was generally high from bloom until around 15 DAFB (peak in expression around 7 DAFB), coincident with the period of rapid cell production. A sharp decline in expression was noted between 15 DAFB and 25 DAFB by ~8-fold and ~3-fold in *MdANT1* and *MdANT2*, respectively, and was coincident with the initial decline in cell production. The expression of these genes declined further between 32 and 39 DAFB, coincident with exit from cell production, and remained low throughout the rest of fruit development. The above data indicate that the expression of *MdANT1*and *MdANT2* was closely associated with cell production during early fruit growth.

In addition to *MdANT1* and *MdANT2*, the expression of five *AILs* was studied during fruit development in ‘Gala’. *MdAIL1*, *MdAIL2*, and *MdAIL3* displayed highest expression primarily before full bloom (Additional file 3). Their expression declined rapidly during early fruit development and remained low throughout the rest of fruit development (Additional file 3). *MdAIL4* and *MdAIL5* also displayed a similar pattern except that the expression of these genes transiently increased by ~6 and ~12-fold, respectively, between 14 to 18 DAFB and was followed by low levels of expression throughout the rest of fruit development (Additional file 3).

### Expression of *MdANT1* and *MdANT2* is enhanced in response to increase in carbohydrate availability

**Figure 4:**
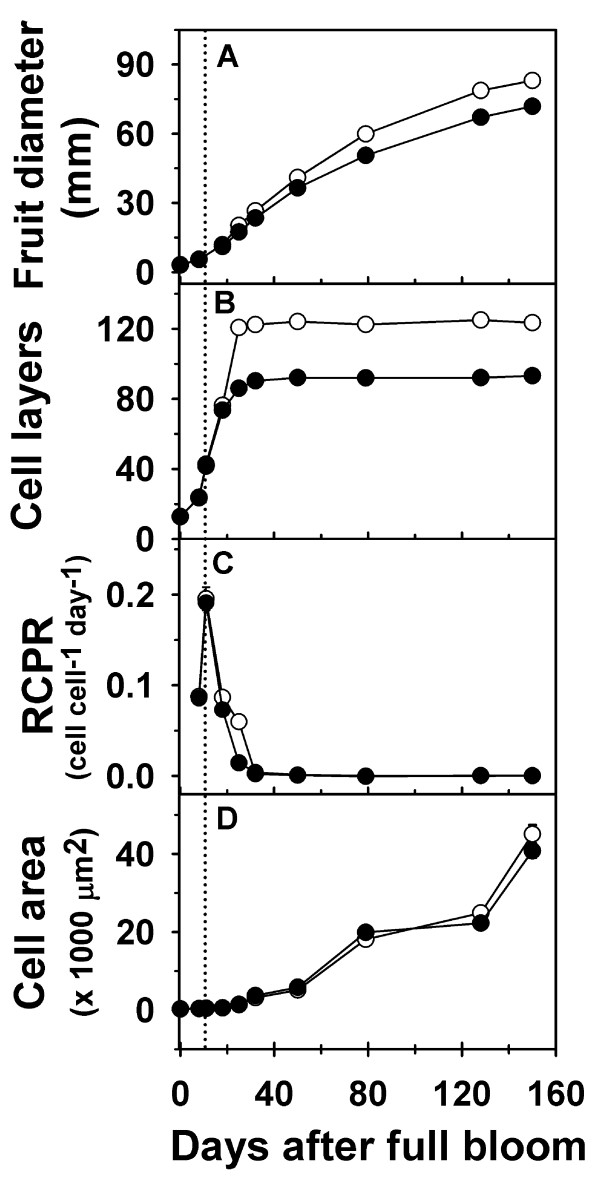
**Fruit growth and cell growth-related parameters during fruit development in thinned and un-thinned fruit of ‘Golden Delicious Smoothee’ (GS).** (**A**) Fruit diameter, (**B**) cell number (layers), (**C**) relative cell production rate (RCPR) and (**D**) cell area were measured from bloom to maturity. Closed circles represent un-thinned fruit and open circles represent thinned fruit. Dotted line represents the day of manual thinning (11 DAFB). Error bars represent the standard error of the mean of four biological replicates (*n* = 4).

In ‘Golden Delicious Smoothee’ (GS), manual thinning at 11 DAFB led to enhanced fruit growth and an increase in fruit size (Figure 4A; Additional file 4). A 16% increase in fruit diameter was observed in thinned fruit by around 25 DAFB (*P* = 0.004), indicating that thinning resulted in a rapid increase in early fruit growth. At maturity, thinned fruit had higher fruit diameter (~16%; *P* < 0.001) and fruit weight (35%; *P* < 0.001) than un-thinned fruit. Enhanced fruit growth during early fruit development in thinned fruit was primarily associated with an increase in cell production in the fruit cortex. Cell production was similar between thinned and un-thinned fruit until around 18 DAFB. The extent of cell production in the fruit cortex was higher in thinned fruit between 18 and 25 DAFB, than that in un-thinned fruit. Cell number in un-thinned fruit was lower than that in thinned fruit by ~30% (*P* < 0.001) at 25 DAFB, and remained lower during the rest of fruit development (Figure 4B). The RCPR was 3-fold higher in thinned fruit at 25 DAFB (Figure 4C). Cell area within the fruit cortex was significantly higher in thinned fruit in comparison to that in un-thinned fruit at 128 DAFB (~12%; *P* = 0.019) and 150 DAFB (~11%; *P* < 0.001; Figure 4D). These data indicate that increase in carbohydrate availability due to thinning enhanced fruit growth primarily by increasing cell production during early fruit growth and cell expansion at later stages.

**Figure 5:**
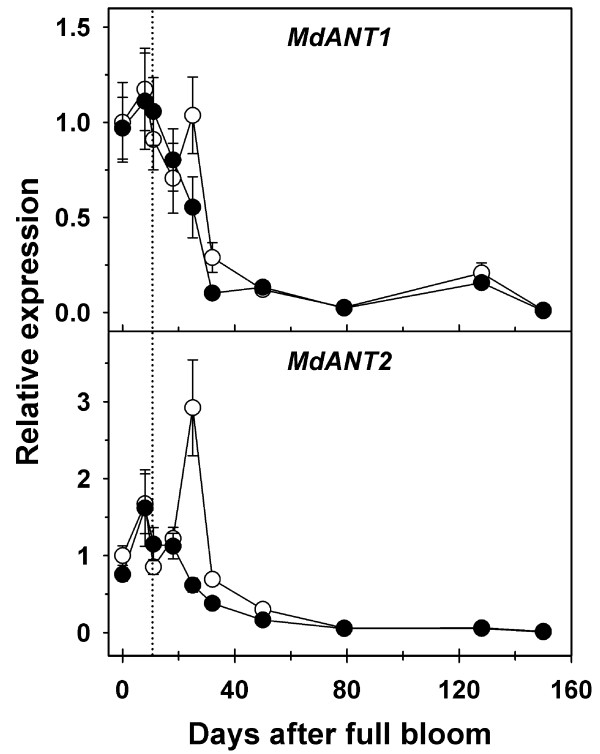
**Expression of*MdANT1*and*MdANT2*in thinned and un-thinned ‘Golden Delicious Smoothee’ (GS) fruit.** Gene expression was analyzed from bloom to maturity. Dotted line represents the day of thinning (11 DAFB). Fold-change in the expression of a gene relative to its expression in thinned fruit at full bloom (0 DAFB) is presented here. *MdACTIN* and *MdGAPDH* were used as the reference genes in this study. Error bar represents the standard error of mean of four biological replicates (*n* = 4). Closed circles represent un-thinned fruit and open circles represent thinned fruit.

Expression of *MdANT1* and *MdANT2* was not significantly different between thinned and un-thinned fruit until after 18 DAFB (Figure 5; Additional file 4). In comparison to un-thinned fruit, expression of *MdANT1* was almost 2-fold higher (*P* = 0.005), while that of *MdANT2* was around 5-fold higher in thinned fruit at 25 DAFB (*P* < 0.001). Expression of *MdANT1* in thinned fruit was also significantly higher at 32 DAFB (*P* < 0.001), while that of *MdANT2* was significantly higher at 32 DAFB (~2-fold; *P* = 0.009) and 50 DAFB (~2-fold; *P* = 0.009). Interestingly, thinning resulted in a transient up-regulation in the expression of *MdANT2*. At 25 DAFB, expression of *MdANT2* was >3-fold and >2-fold higher than that at 11 and 18 DAFB respectively, in thinned fruit. Together, the above data indicate that enhanced expression of *MdANT1* and *MdANT2* due to thinning was associated with an increase in cell production.

### *MdANT1* and *MdANT2* are differentially expressed across different fruit size genotypes during early fruit growth

**Figure 6:**
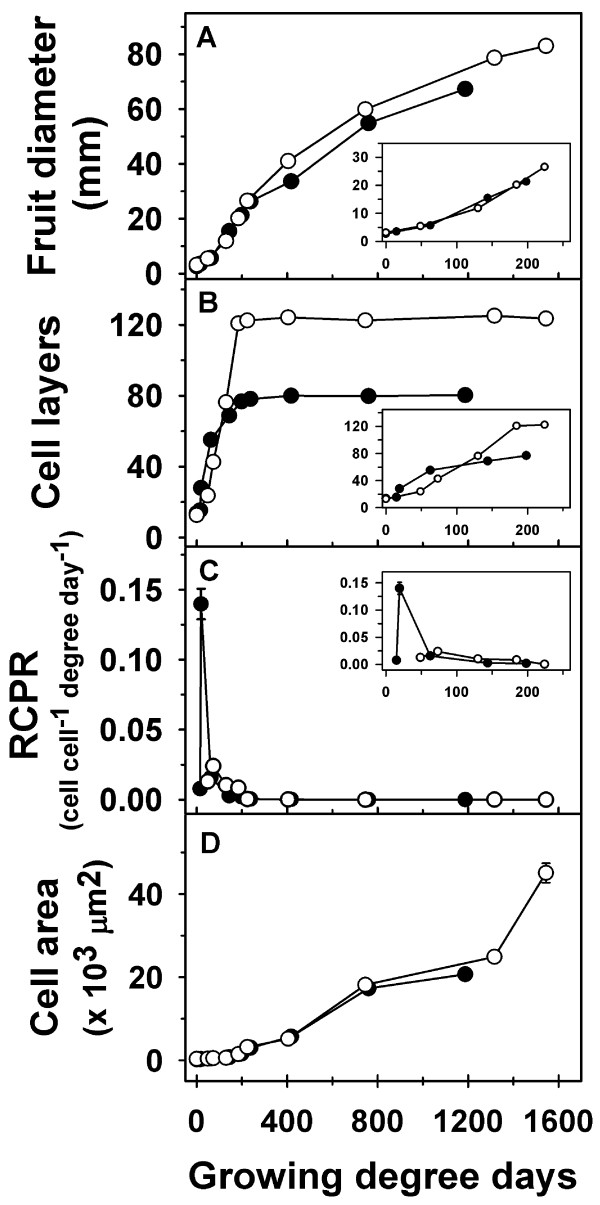
**Comparison of fruit growth and cell growth-related parameters during fruit development in ‘Gala’ and ‘Golden Delicious Smoothie’ (GS).** (**A**) Fruit diameter, (**B**) cell number (layers), (**C**) relative cell production rate, and (**D**) cell area were measured from bloom until maturity. Closed circles represent ‘Gala’ and open circles represent GS. Error bar represents the standard error of the mean of four biological replicates (*n* = 4). Data presented here are from Figures 2 and 4 (thinned fruit only). Cumulative growing degree days after full bloom (GDD) were used to allow for comparison across the two genotypes. Insets display changes in fruit diameter, cell number and RCPR during early fruit development.

Fruit growth and development were compared across ‘Gala’, a medium fruit size genotype, and GS, a large fruit size genotype. ‘Gala’ flowers were in full bloom ~7 days prior to that of GS. Hence, growing degree days after bloom (GDD) were used to allow for comparison of fruit growth, cell production and gene expression parameters between ‘Gala’ and GS. Both genotypes displayed a similar pattern of fruit growth, except that GS had a longer growing period of 1544 GDD in comparison to 1187 GDD in ‘Gala’ (Figure 6A; Additional file 5). The initial phase of fruit growth in ‘Gala’ involved a rapid increase in fruit diameter which continued until around 237 GDD after which fruit diameter increased linearly until fruit maturity. In GS, the initial phase of rapid fruit growth continued for a longer period (around 404 GDD) after which fruit diameter increased linearly until maturity. Final fruit diameter in GS was 23% higher than that in ‘Gala’. Both genotypes displayed a similar number of cell layers within the floral-tube at full bloom (0 GDD; Figure 6B inset). ‘Gala’ and GS displayed differences in the pattern of progression in cell production within the fruit cortex. In ‘Gala’, cell number within the fruit cortex increased rapidly until around 62 GDD, continued to increase at a lower rate between 62 and 198 GDD, and remained largely unchanged thereafter. In GS, increase in cell number within the fruit cortex was observed from around 48 GDD until around 184 GDD after which it remained largely unchanged (Figure 6B inset). Cell number at maturity in GS was almost 54% higher than that in ‘Gala’. The RCPR maxima in ‘Gala’ was around 0.14 cell cell^-1^ GDD^-1^,while that in GS was around 0.024 cell cell^-1^ GDD^-1^ (Figure 6C). However, the peak in RCPR in ‘Gala’ was attained around 19 GDD while that in GS was attained at around 73 GDD (Figure 6C). Potentially higher RCPR levels were maintained in GS than that in ‘Gala’ from around 73 GDD until the end of the cell production period. Final area of the fruit cortex cells in GS was around 2-fold higher in GS than that in ‘Gala’ (Figure 6D).

**Figure 7:**
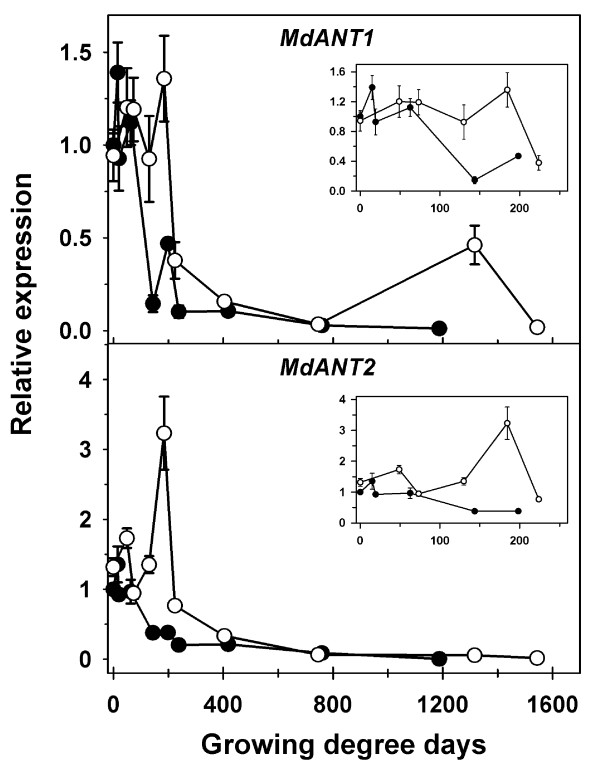
**Comparison of expression of*MdANT1*and*MdANT2*during fruit development in ‘Gala’ and ‘Golden Delicious Smoothie’ (GS).** Closed circles represent ‘Gala’ and open circles represent GS. Data presented here are from Figures 3 and 5 (thinned fruit only). Cumulative growing degree days after full bloom (GDD) were used to allow for comparison of the two genotypes. Fold-change in the expression of a gene relative to its expression at full bloom (0 DAFB) in ‘Gala’ is presented. Error bar represents the standard error of mean of four biological replicates (*n* = 4). The reference genes *MdACTIN* and *MdGAPDH* were used in this study. Insets display the expression of the genes during early fruit development.

*MdANT1* expression in ‘Gala’ increased after bloom but was subsequently similar to that in GS until around 62 GDD (Figure 7 inset; Additional file 5). After 62 GDD, transcript abundance of *MdANT1 *declined rapidly in ‘Gala’ but continued to remain high in GS until around 184 GDD. Around this period, *MdANT1* expression was substantially higher in GS (3- to 10-fold) in comparison to that in ‘Gala’. Expression of *MdANT2* appeared to be slightly higher in GS than that in ‘Gala’ until around 48 GDD (Figure 7 inset; Additional file 5). Between 62 and 198 GDD, the expression of *MdANT2* declined in ‘Gala’ by around 3-fold. During a similar period (73-184 GDD) the expression of *MdANT2* in GS increased by >3-fold. Around 184 GDD, *MdANT2* expression was ~6-fold higher in GS in comparison to that in ‘Gala’. At later stages of fruit development, a period of post mitotic cell expansion-mediated growth, the *ANTs* displayed very low levels of expression in both the genotypes.

### *MdANT1* and *MdANT2* are differentially expressed between the core and the cortex

Apple is an accessory fruit (pome) where the floral-tube tissue surrounding the ovary develops into the fleshy and edible region of the fruit (cortex) while the ovary develops into the core [37]. Laser capture microdissection (LCM) was used to isolate these tissues and the localization of *MdANT1* and *MdANT2* expression within these tissues during early fruit development was studied. Expression of two MADS box genes, *MdMADS5* and *MdMADS10*, was also analyzed. Previous research indicated that *MdMADS5* was predominantly expressed in the cortex and the skin while *MdMADS10* was primarily expressed in the core tissue [38]. In the present study, *MdMADS5* expression was clearly higher in the cortex tissue than in the core by 2.5- to 11-fold during different stages of flower development and early fruit growth (-7 to 15 DAFB; Additional file 6). Also, the expression of *MdMADS10* was consistently higher in the core tissue than in the cortex by 5- to 9-fold between -7 and 15 DAFB (Additional file 6). These data are consistent with the previous report [38], and demonstrate that the cortex and core tissues were effectively isolated using LCM.

**Figure 8:**
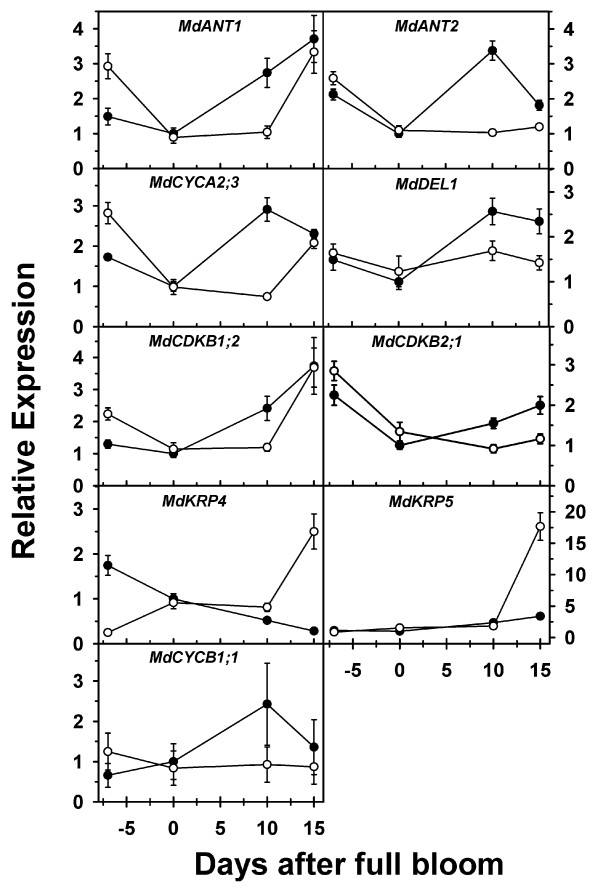
**Expression of*MdANT1, MdANT2*and cell cycle genes in the core and cortex of ‘Gala’ during flower and early fruit development.** Open circles represent the core tissue (ovary at -7 DAFB) while closed circles represent the cortex tissues (floral-tube at -7 DAFB). These tissues were isolated using laser capture microdissection. Gene expression was determined using qRT-PCR and was normalized using *MdACTIN*. Expression of a gene relative to its expression at full bloom (0 DAFB) is presented here. Error bar represents the standard error of the mean of three biological replicates (*n* = 3).

*MdANT1* and *MdANT2* were expressed in the core as well as the cortex tissues during different stages of flower and early fruit development. Expression of these genes in both tissues was high before bloom and declined by up to 3-fold between -7 DAFB and full bloom (Figure 8). *MdANT1* expression was almost 2-fold higher in the ovary tissue than the floral-tube at -7 DAFB. Between bloom and 10 DAFB, *MdANT1* and *MdANT2* expression increased by 3-fold and 4-fold respectively in the cortex tissue, while it remained largely unaltered in the core. At 15 DAFB, expression of *MdANT2* within the cortex continued to be higher than that in the core, but the expression of *MdANT1* in the core reached levels similar to that in the cortex.

### *MdANT1* and *MdANT2* expression is correlated with that of cell cycle genes

Expression of several cell cycle genes was investigated in the core and cortex tissues isolated using LCM (Figure 8). Genes positively associated with cell production such as *MdCYCA2;3MdCYCB1;1MdCDKB1;2MdCDKB2;1* and *MdDEL1*[1] displayed expression patterns similar to that of *MdANT1* and *MdANT2* during early fruit growth and development. At 10 DAFB, expression of these genes was up to 3-fold higher in the cortex in comparison to that in the core. *MdANT1* and *MdANT2* expression was significantly correlated with that of *MdCYCA2;3MdCYCB1;1MdCDKB1;2MdCDKB2;1* and *MdDEL1* [R = 0.86, 0.49, 0.75, 0.58, and 0.68 (with *MdANT1*); 0.83, 0.62, 0.45, 0.73 and 0.69 (with *MdANT2*), respectively]. *MdKRP4*, a gene negatively associated with cell production, displayed a gradual increase in expression in the core tissue during early fruit growth while it declined steadily within the cortex. At 15 DAFB, *MdKRP4* displayed ~10-fold higher expression in the core in comparison to that in the cortex. *MdKRP4* expression was not significantly correlated with either of the apple *ANT* genes. *MdKRP5*, another gene negatively associated with cell production, displayed a minor increase in expression until 10 DAFB in the core and the cortex. At 15 DAFB, *MdKRP5* expression increased greatly (~10-fold) within the core but only slightly in the cortex, resulting in >6-fold difference in expression between these tissues. *MdANT1* expression was weakly correlated with that of *MdKRP5* (R = 0.51; *P* = 0.01).

## Discussion

Several lines of evidence from this study suggest that *MdANT1* and *MdANT2* function as transcription factors in apple. A motif of basic residues (KKKR) is essential for the nuclear localization of ANT, as replacement of two lysine residues within this motif resulted in a loss of nuclear localization in Arabidopsis [36]. In MdANT1 and MdANT2, a major part of this element is conserved (TKKR), strongly suggesting that these ANTs are targeted to the nucleus, consistent with their proposed roles as transcription factors. The Arabidopsis ANT binds to the DNA at a consensus site of 16 bases through two AP2 domains and a conserved linker region [24]. MdANT1 and MdANT2 shared greater than 88% sequence identity with the Arabidopsis ANT within these regions. All of the 19 residues identified as essential for the DNA binding activity of the Arabidopsis ANT [25] are conserved in the apple ANTs suggesting that they may bind to similar DNA elements, further supporting their role as transcription factors. Domains within the amino-terminal region are also essential for the transcriptional activation properties of the Arabidopsis ANT [36]. Although the apple ANTs display limited conservation of residues with that of the Arabidopsis ANT in this region, it should be noted that other plant ANTs also display significant sequence divergence within this region, indicating that distinct, species-specific features may be required for the transcriptional activation properties of the ANTs.

*MdANT1* and *MdANT2* are expressed in regions associated with fruit growth and development [a) ovary and floral-tube tissues before bloom; b) core and cortex tissues during early fruit growth]. *MdANT1* and *MdANT2* display high expression before bloom in the ovary as well as the floral-tube regions, strongly suggesting their association with cell production-mediated growth of the ovary and floral-tube tissues before bloom. Expression of *MdANT1* and *MdANT2* declines within these tissues during the period of temporary cessation of growth and quiescence in cell production (around full bloom). Subsequently, the expression of the *ANT*s increases sharply within the cortex tissue while little change in their expression is observed within the core tissue between bloom and 10 DAFB, coincident with the resumption of growth and re-initiation of cell production in the cortex during early fruit development. The sharp increase in expression at 10 DAFB within the cortex is likely triggered by pollination and/or fertilization and may mediate fruit set. *MdANT1* and *MdANT2* expression is high during the cell production-mediated phase of early fruit growth and subsequently declines greatly during exit from this phase. This pattern of expression is conserved under conditions of different carbohydrate availability and across genotypes differing in their fruit growth potential. Together, the data presented here indicate that the expression of *MdANT1* and *MdANT2* is consistently and closely associated with cell production during fruit growth in apple. Therefore, it is proposed that *ANTs* are important components of a developmental program that controls the extent of cell production and thereby regulates fruit growth in apple.

Cell production and fruit growth are limited by carbohydrate availability in many plant species [4,39-42]. Consistent with previous studies, increase in carbohydrate availability through manual thinning during early fruit development in GS enhanced fruit growth and final fruit size. This was primarily achieved through sustained cell production in the fruit cortex during early fruit growth and a higher relative cell production rate, especially towards the later stages of the cell production phase. These data indicate that carbohydrate limitation due to increased competition among sinks decreases the extent of cell production in the fruit cortex. Under conditions of higher carbohydrate availability, the expression of *MdANT1* and *MdANT2* was several-fold higher than that under carbohydrate limitation. Additionally, *MdANT2* was up-regulated (>3-fold at 25 DAFB compared to 11 DAFB) in response to an increase in carbohydrate availability in GS. These data suggest that an increase in carbohydrate availability enhances the expression of the *ANT* genes, especially *MdANT2*, thereby increasing the competence of the fruit cortex cells for cell production. Hence, it may be proposed that the *ANTs*, particularly *MdANT2*, mediate the effects of carbohydrate availability on cell production and fruit growth. Increase in competence for cell production may be achieved either through an increase in the proportion of fruit cortex cells undergoing proliferation or through an increase in the capacity of individual cortex cells for division. Increase in carbohydrate availability also led to a minor increase in cell area during later stages of fruit growth in GS, inconsistent with previously reported results in the apple cultivar, ‘Empire’ [4], but consistent with results in tomato fruit [39,42]. It is likely that an increase in sink strength as a result of higher fruit cortex cell number in thinned fruit may subsequently aid in increasing the extent of cell expansion.

Comparison of apple genotypes differing in their growth potential further supports the proposed roles of the *ANTs* in regulating cell production. Although, it is possible that some of the differences observed between the two genotypes are due to environmental effects, the overall patterns of fruit growth and gene expression reported here were consistent with that observed in other studies during different years (data not shown). The initial cell number and the duration of the cell production phase were similar in ‘Gala’ and GS (around 198 and 184 GDD after bloom, respectively), indicating that the higher final cell number within the fruit cortex of GS in comparison to that in ‘Gala’ was due to differences in the pattern of progression in cell production during early fruit development. GS fruit cortex cells displayed a more gradual increase in cell number after bloom in comparison to those of ‘Gala’ which displayed a short-lived early burst in cell production. In fact, the RCPR in GS reached the maxima around 54 GDD after that in ‘Gala’. Subsequently, the rate of cell production in GS was higher than that in ‘Gala’, especially between 73 and 184 GDD after bloom. Expression of *MdANT1* and *MdANT2* in the two genotypes matched their respective patterns of cell production. Expression of these genes in GS was sustained at higher levels for a longer duration while in ‘Gala’, the expression of these genes displayed an initial rapid burst followed by a rapid decline. The expression of both these genes was higher in GS than in ‘Gala’ during the final stages of the cell production phase (around 129 and 184 DAFB). Sustained competence for cell production as a result of this pattern of expression of the *ANTs* may allow for enhanced cell production and a higher final cell number in GS. Final cell number is often an important determinant of variation in fruit size across genotypes [5,9,43]. Differences in the pattern of expression of the *ANT* genes during early fruit growth may affect the final cell number and thereby final fruit size across genotypes. Similarly, differences in the pattern of expression of *FW2.2* are thought to determine fruit size differences across tomato genotypes [44]. Hence, it is likely that *MdANT1* and *MdANT2* also function as regulators of fruit size in apple.

Expression of the apple *ANT* genes was correlated with that of several positive regulators of the cell cycle, including B-type CDKs, A- and B-type cyclins, and *MdDEL1* during different stages of flower and early fruit development. During the period of exit from cell production (around 15-25 DAFB in ‘Gala’), the expression of several cell cycle genes positively associated with cell production declined, while that of genes negatively associated with cell production increased [1]. These changes in the expression of the cell cycle genes coincide with the decline in the expression of *MdANT1* and *MdANT2* observed in this study. In fact, the expression patterns of the *ANT* genes during fruit growth display high similarity with those of the core cell cycle genes involved primarily in the regulation of the G2-M phases of the cell cycle. Co-expression of these genes suggests coordinated regulation and their involvement in a common biological process [45]. Considering that the *ANT* genes may function as transcription factors, it is possible that *MdANT1* and *MdANT2* regulate the expression of the core cell cycle genes and thereby coordinate cell production during fruit growth. In Arabidopsis, increased cell production as a result of the over-expression of *ANT* was associated with the prolonged expression of D3-type cyclins [19]. Identification of the genes targeted for direct regulation by the ANTs is essential to test this hypothesis.

The general similarities in the expression patterns of *MdANT1* and *MdANT2* suggest overlapping roles for these genes in regulating flower and fruit development. In Arabidopsis, expression of four *PLT* genes (members of the AP2 sub-family) in overlapping as well as specific regions of the root allows for PLT concentration-dependent regulation of root growth and development [32]. Similarly in apple, the combined activity of MdANT1 and MdANT2 may have an additive effect on cell production and fruit growth. However, certain key differences between *MdANT1* and *MdANT2* were also noted. The expression of these genes in the core tissue differed slightly during early fruit development. MdANT1 and MdANT2 also differed within the AP2-repeats and linker region in three residues (A354-S352; T365-A363; S388-F386, MdANT1-MdANT2, respectively). If the DNA binding characteristics are affected by the above residues, MdANT1 and MdANT2 may regulate different pools of downstream target genes. Together, the above data suggest that *MdANT1* and *MdANT2* may also have distinct roles in regulating fruit growth and development. Functional characterization of *MdANT1* and *MdANT2* and the identification of their downstream targets *in vivo* are essential to determine their specific roles in regulating fruit growth.

All of the *AIL* genes studied here contained the characteristic AP2-repeats and the conserved linker region suggesting that they function as transcriptional regulators. These genes displayed elevated expression during flower development and a sharp decline in expression during early fruit development, suggesting that they may be primarily involved in regulating flower growth and development in apple. In Arabidopsis, many of the *AIL* genes are involved in regulating floral organ growth and development [21,34,35]. MdAIL4 and MdAIL5 share significant amino acid identity with AtAIL5 and AtAIL6 respectively, genes which have been previously reported to regulate organ growth [21,34,35]. Further characterization of the tissue-specific patterns of expression and the functional characterization of the *AIL* genes is essential to determine their specific roles in apple.

## Conclusions

Data presented here strongly suggest that *MdANT1* and *MdANT2* regulate cell production and fruit growth in apple by coordinating the expression of genes involved in cell proliferation. *MdANT1* and *MdANT2* are a significant addition to the limited list of candidate upstream regulatory genes involved in the control of growth of fleshy fruit. Functional characterization of these genes and the identification of their downstream targets may greatly aid in unraveling the mechanisms involved in the regulation of fruit growth in apple and other fleshy fruit.

## Methods

### Plant material

Mature ‘Gala’ and ‘Golden Delicious Smoothee’ (GS) trees growing on M.7 and M.7a rootstocks respectively, at the Georgia Mountain Research and Experiment Station in Blairsville, GA, USA were used in this study. Fruit growth and development was studied using four randomly selected ‘Gala’ trees at the above location in 2009. Each of these trees was treated as an independent replicate (*n* = 4). Trees were manually thinned to one lateral fruit per cluster at 10 DAFB. Fruit diameter was measured from bloom until maturity on 20 fruit per replicate. At each stage, fruit were randomly sampled from different parts of the canopy between 12 pm and 2 pm, independently from each replicate. At each stage, four fruit from each replicate were fixed in CRAF III fixative for cytology. At each stage, fruit tissue from at least four fruit was pooled within each replicate and frozen in liquid N_2_ for gene expression analyses. To determine the affect of carbohydrate availability on fruit growth, four randomly selected GS trees were subjected to the thinning treatment while four other trees were left un-thinned in 2009. Each tree was treated as an independent replicate (*n* = 4). Thinning involved the manual removal of all fruit within a cluster except for one lateral fruit at 11 DAFB. Fruit diameter was measured on 20 fruit per replicate from bloom until maturity. Fruit were sampled at different stages of development for cytology and gene expression analyses as described above. All trees used in the above studies were maintained according to commercial apple production practices except for the application of chemical thinning agents.

In 2010, three ‘Gala’ trees, each of which was treated as an independent replicate (*n* = 3), were used to determine the localization of expression of several genes using laser capture microdissection (LCM). For this study, lateral flowers/fruit were randomly sampled from different parts of the tree canopy at -7, 0, 10 and 15 DAFB. At least four individual flowers/fruit from each replicate were used at each stage in this experiment. All sampling was performed between 12 pm and 2 pm. The ovary and floral-tube tissues, or the fruit was dissected and fixed in freshly prepared Farmer’s fixative containing 75% (v/v) ethanol and 25% (v/v) acetic acid, and stored at 4°C until further analysis. Manual thinning or application of chemical thinning agents was not performed in this study.

### Measurement of cell number and cell area

Cell number and cell area were determined as described previously [1]. Briefly, sectioning of flower/fruit was performed using a vibratome (Micro-cut H1200, Bio-Rad, Hercules, CA, USA). Cell number was determined by counting the number of cell layers between the petal vascular trace and the epidermis in sections stained with toluidine blue. The relative cell production rate (RCPR) was determined from the cell number data as: *Ln*(C_2_)–*Ln*(C_1_)]/T_2_–T_1_, where C_1_ and C_2_ denote the cell number at time points T_1_ and T_2_, respectively. To measure the cell area, the number of cells within a defined area was determined at three locations between the epidermis and the petal vascular trace. Cell area was calculated using this value and the average cell area from the three locations was used as the cortex cell area of the fruit sample.

Comparison of various parameters such as fruit growth, cell number, cell area and gene expression was performed across the genotypes, ‘Gala’ and GS. Data from the fruit development study in ‘Gala’ and the thinning study in GS (only thinned fruit) described above were used for this comparison (2009). As the genotypes differed significantly in terms of the time of full bloom (by around 1 week), cumulative growing degree days (GDD) from the time of the respective full bloom dates were used to allow for this comparison. GDD was determined using temperature data obtained from the Georgia weather network (http://www.georgiaweather.net). A base temperature of 10°C was used for GDD determination. If the average daily temperature was below 10°C, GDD accumulation was set to zero [46].

### Isolation of the apple *ANT* genes

Publicly available apple expressed sequence tag (EST) database (National Center for Biotechnology Information-NCBI) was mined to identify genes with homology to the Arabidopsis *ANT* (*AtANT*; [GenBank:At4G37750]). Eight potential genes with similarity to the *AtANT* and other *AIL* genes were identified. The EST displaying highest homology to *AtANT* was designated as *MdANT*. Preliminary gene expression analyses were performed to determine the pattern of expression of these genes during apple fruit development. Expression analyses was performed using fruit collected from mature ‘Gala’ trees in 2008 (*n* = 4; previously described in [1]). *MdANT* displayed higher expression during the cell production phase of fruit development and was selected for further analysis.

To isolate the full-length cDNA of the *MdANT* gene, 5′ and 3′ RACE (Rapid Amplification of cDNA Ends) were attempted. Total RNA was extracted from ‘Gala’ fruit at 10 DAFB as the gene displayed high expression at this stage in the preliminary analysis. First strand cDNA synthesis and amplification were performed using the SMART RACE cDNA Amplification kit (Clontech Laboratories Inc., CA, USA) following the manufacturer’s instructions. The 5′ and 3′ gene-specific primers for this analysis were designed using the EST sequences of *MdANT*. The 3′ RACE analysis of *MdANT* yielded two products which were subsequently cloned into the pGEM-T Easy vector (Promega Corporation, WI, USA) and sequenced. The 3′ RACE products displayed high homology (>90% identity) with each other, and were designated as *MdANT1* and *MdANT2*. Several attempts were made to isolate the 5′ sequences of *MdANT1* and *MdANT2*. Techniques such as 5′ RACE and degenerate PCR using primers designed from the highly conserved regions of multiple *ANT* genes {*Arabidopsis thaliana* (*AtANT*; [GenBank:ABR21533]), *Vitis vinifera* (*VvANT*; [GenBank:AM444297]), *Brassica napus* (*BnANT*; [GenBank:ABA42146]), *Populus trichocarpa* (*PtANT*; [GenBank:AC210555]), *Nicotiana tabaccum* (*NtANT*; [GenBank:AAR22388]), *Artemisia annua* (AaANT; [GenBank:ACY74336])} were used. However, these attempts were largely unsuccessful. Following the release of the peach (*Prunus persica*) genome, primers were designed using the peach *ANT* (*PpANT*; [Genome database for Rosaceae:ppa023077m]). The 5′ sequence of *MdANT2* was amplified, cloned and sequenced using this approach. Following the release of the apple genome [47], *MdANT1* and *MdANT2* were identified from the apple genome database (http://genomics.research.iasma.it) using the sequence information derived from the above approaches. Primers were designed for full-length amplification of *MdANT1* and *MdANT2*. The PCR amplified products were cloned into pGEM-T Easy vector (Promega Corporation, WI, USA) and sequenced. Accession numbers for *MdANT1* and *MdANT2* are MDP0000175309 and MDP0000190889, respectively [Apple genome database (http://genomics.research.iasma.it)]. Sequence of the above genes obtained in this study differed from the predicted sequence available in the apple genome database primarily with respect to the presence of a ‘VYL’ motif within the DNA binding domain. Primer sequences used in the above approaches for cloning the apple *ANT* genes are provided in the Additional file 7.

### Phylogenetic analysis

Plant ANT sequences were retrieved from the NCBI database, Genome Database for Rosaceae and The Arabidopsis Information Resource (TAIR). Multiple alignments of apple and other plant ANT transcription factors were performed using MUSCLE (Multiple Sequence Comparison by Log-Expectation; http://www.ebi.ac.uk/Tools/msa/muscle/). Phylogenetic tree construction was performed using the neighbor joining distance method of the MEGA5 (Molecular Evolutionary Genetics Analysis) software [48].

### RNA extraction and quantitative reverse transcription-PCR (qRT-PCR)

RNA extraction from flower and fruit was performed using the method described previously [3], except that the extraction buffer contained 150 mM Tris-HCl instead of Tris-Borate. The cDNA synthesis was performed as described previously [1] using 1 μg of total RNA after removal of genomic DNA with a DNase treatment (Promega Corporation, WI, USA). Reverse transcription was performed using ImProm II reverse transcriptase (Promega Corporation, WI, USA) and oligo dT (15) primers. The cDNA was diluted 5-fold for all gene expression analyses. Gene-specific primers for qRT-PCR analyses of *MdANT1* and *MdANT2* were designed from regions sharing low homology and are shown in Additional file 8. Primer efficiency was determined for the primer pairs and ranged from 1.85 to 1.97. The 2X SYBR GREEN master mix (Applied Biosystems, Carlsbad, CA, USA) was used for all analyses. All the qRT-PCR analyses were performed using the Stratagene Mx3005P real-time PCR system as described previously [1]. Briefly, the reaction conditions were as follows: 95°C for 10 min; 40 cycles of 95°C (30 s) and 60°C (1 min). Melt-curve analyses were performed after the PCR. A single distinct peak was observed for all the genes studied indicating the specific amplification of a single product. No-template controls were included in each run of the qRT-PCR. Relative expression was calculated using a modified Pfaffl method [49] and as described in [50]. Relative quantity (RQ) for each sample was calculated using the formula, 1/E^Cq^, where Cq is the quantification cycle (threshold cycle). The RQ was normalized using two reference genes, *MdACTIN* and *MdGAPDH* (accession numbers [Genbank:EB127077] and [Genbank:EB146750], respectively; described previously in [1]). The geometric mean of expression of the two reference genes (normalization factor) was used for normalization. The normalized RQ (NRQ) values were log_2_ transformed and used for statistical analyses. The standard error of the means was calculated as described in [50]. Expression of a gene relative to its expression at full bloom (0 DAFB) is presented for the fruit development study in ‘Gala’. For the thinning study in GS, expression of a gene relative to its expression at full bloom (0 DAFB) in thinned fruit is presented. For the study involving comparison of gene expression between ‘Gala’ and GS, expression of a gene relative to its expression at 0 DAFB in ‘Gala’ is presented. In all the above studies, four independent biological replicates were used for the qRT-PCR analyses.

### Laser capture microdissection (LCM)

Flower (or fruit) sampled at -7, 0, 10 and 15 DAFB and fixed in Farmer’s fixative were rehydrated in a graded series of ethanol (2 h each in 75%, 50%, 30% and 0% ethanol prepared with DEPC-treated water) at 4°C. The samples were embedded in 6% agarose (prepared in DEPC-treated water) and sectioned using a vibratome. All surfaces of the vibratome were cleaned with RNaseZAP solution (Ambion, Inc., TX, USA) and rinsed with DEPC-treated water before use. The sections were placed on a glass slide and LCM was performed using the PALM MicroBeam system (Carl Zeiss Microscopy, LLC, NY, USA). LCM was performed with the laser beam set to a power of 60 to 90 mW. Microdissected cells were collected in the lid of a 0.6 mL reaction tube containing the RNA extraction buffer (150 mM Tris-HCl, 50 mM EDTA, 2% SDS, and 1% β-mercaptoethanol). The microdissected cells from flowers/fruit within a replicate were pooled for RNA extraction. Captured tissues were transferred to a tube containing the extraction buffer followed by the addition of PVPP. To this mix, 0.1 volumes of 5 M potassium acetate and 0.25 volumes of ethanol were added and the mixture was extracted with chloroform:iso-amyl alcohol (24:1 v/v), followed by extraction with phenol:chloroform:iso-amyl alcohol (25:24:1 v/v) and chloroform:iso-amyl alcohol (24:1 v/v). The aqueous supernatant was precipitated with isopropanol (1:1 v/v) at room temperature for 15 min, followed by precipitation in 3 M lithium chloride (4°C) for 2 h. RNA was subsequently washed with 70% ethanol, air dried, and dissolved in DEPC-treated water. Total RNA (0.5 μg) was used for cDNA synthesis. cDNA synthesis and and qRT-PCR analyses were performed as described above. Only *MdACTIN* was used as the reference gene as *MdGAPDH* did not display stable expression across the samples in this study. Calculation of gene expression was performed as described above. The cell cycle genes, *MdCYCA2; 3* [Genbank:CO415585], *MdCYCB1;1* [Genbank:CN579062], *MdCDKB1;2* [Genbank:EB138473], *MdCDKB2;1* [Genbank:CV129014], *MdDEL1* [Genbank:CV631574], *MdKRP4* [Genbank:CV084380] and *MdKRP5* [Genbank:CN912198] were used in this study and have been described previously [1]. *MdMADS5* (Apple Genome Database:MDP0000013331) and *MdMADS10* [Genbank:AJ000762] were used to confirm the isolation of core and cortex tissues by LCM. Primer sequences for the two *MdMADS* genes are provided in Additional file 8. Expression of a gene relative to its expression at 0 DAFB in the cortex tissue is presented here. Three independent biological replicates were used for the qRT-PCR analysis.

### Statistical analysis

Statistical analyses were performed using SAS 9.0 (SAS Institute Inc., NC, USA) and SigmaPlot 11 (Systat Software Inc., San Jose, CA). Fruit diameter, cell layers, cell area and qRT-PCR data were compared between thinned and un-thinned treatments using two-way ANOVA. The paired *t* test was used for statistical comparison of gene expression between the core and cortex tissues isolated by LCM. Pearson product moment correlation analysis was used to analyze the association between the expression of *MdANT1*, *MdANT2* and the cell cycle genes. NRQ values (log_2_ transformed) were used for the above analyses.

## Competing interests

The authors do not have any competing interests.

## Authors’ contributions

MD and AM conceived and designed this research. MD executed all the experiments. AM supervised the research. MD and AM performed data analyses and drafted the manuscript. All authors read and approved the final manuscript.

## Supplementary Material

Additional file 1 Comparison of the predicted amino acid sequences of plant ANTs. (A) Phylogenetic analysis of two apple ANTs, and Arabidopsis ANT and AILs was performed using the neighbor joining distance method of MUSCLE. Sequences for Arabidopsis ANT and AILs were retrieved from the NCBI database. The accession numbers for Arabidopsis AILs are: AtAIL1 (AT1G72570); AtAIL2 (AT5G17430); AtAIL3 (AT3G20840); AtAIL4 (AT1G51190); AtAIL5 (AT5G57390); AtAIL6 (AT5G10510); AtAIL7 (AT5G65510) (B) Phylogenetic analysis of two ANTs and five AILs from apple. The apple AIL sequences were retrieved from the apple genome database. The accession numbers for the apple AILs are: AIL1 (MDP0000178745); AIL2 (MDP0000801540); AIL3 (MDP0000121984); AIL4 (MDP0000277643); AIL5 (MDP0000211931). (C) Phylogenetic analysis of ANTs from apple and other plants. Sequences for the ANTs used here were retrieved from the NCBI database and Genome Database for Rosaceae. *Arabidopsis thaliana* (AtANT; ABR21533)*, Brassica napus* (BnANT; ABA42146)*, Artemisia annua* (AaANT; ACY74336), *Triticum aestivum* (TaANT; AB458518.1), *Oryza sativa* (OsANT; AK106306.1), *Sorghum bicolor* (SbANT; XM_002468181.1), *Hordeum vulgare* (HvANT; AK375318.1), *Malus × domestica* (MdANT1), *Malus × domestica* (MdANT2), *Prunus persica* (PpANT; ppa023077m), *Fragaria* × *ananassa* (FaANT; scf0512968), *Nicotiana tabacum* (NtANT; AAR22388), *Vitis vinifera* (VvANT; AM444297)*, Populus trichocarpa* (PtANT; AC210555). Click here for file

Additional file 2 Growth and gene expression during fruit development in ‘Gala’. The table displays data corresponding to Figure 2 for Fruit diameter (Figure 2A), Cell layers (Figure 2B), Relative cell production rate (RCPR; Figure 2C) and Cell area (Figure 2D). The table also displays expression data for *MdANT1* and *MdANT2* from Figure 3. Fruit diameter was not measured at 10 days after full bloom (DAFB). RCPR data were rounded off to the third decimal point. Gene expression was normalized using *MdGAPDH* and *MdACTIN*. Expression of a gene relative to its expression at 0 DAFB is presented. The mean and standard error of four biological replicates are displayed. Click here for file

Additional file 3 Expression of the *AIL* genes during fruit development in ‘Gala’. The normalization factor was determined as the geometric mean of expression of *MdGAPDH* and *MdACTIN*. Fold change in expression is presented relative to expression during full bloom. Error bar represents the standard error of the mean of four biological replicates (*n* = 4). Click here for file

Additional file 4 Growth and gene expression in thinned [A] and un-thinned fruit [B] of ‘Golden Delicious Smoothee’. The table shows data corresponding to Figure 4 for Fruit diameter (Figure 4A), Cell layers (Figure 4B), Relative cell production rate (RCPR; Figure 4C) and Cell area (Figure 4D). The table also shows expression data for *MdANT1* and *MdANT2* from Figure 5. Fruit diameter was not measured at 11 days after full bloom (DAFB). RCPR data were rounded off to the third decimal point. Expression of a gene is presented relative to its expression at 0 DAFB in thinned fruit. Gene expression was normalized using *MdGAPDH* and *MdACTIN*. The mean and standard error of four biological replicates are presented. Click here for file

Additional file 5 Fruit growth and gene expression in ‘Gala’ [A] and ‘Golden Delicious Smoothee’ [B]. The table displays data corresponding to Figure 6 for Fruit diameter (Figure 6A), Cell layers (Figure 6B), Relative cell production rate (RCPR; Figure 6C) and Cell area (Figure 6D). Fruit diameter was not measured at 19 growing degree days (GDD) after full bloom in ‘Gala’ and at 73 GDD after full bloom in ‘Golden Delicious Smoothee’. RCPR data was rounded off to the third decimal point. The table also shows expression data for *MdANT1* and *MdANT2* from Figure 7. Expression of a gene is presented relative to its expression at 0 GDD in ‘Gala’. Gene expression was normalized using *MdGAPDH* and *MdACTIN*. Data for ‘Golden Delicious Smoothee’ are from thinned fruit only. The mean and standard error of four biological replicates are presented here. Click here for file

Additional file 6 Expression of *MdMADS5* and *MdMADS10* in the core and the cortex tissues of ‘Gala’ apple during flower and early fruit development. Closed circles represent cortex tissues and open circles represent core tissues. Core and cortex tissues were separated using laser capture microdissection. Gene expression was determined using quantitative RT-PCR and was normalized using *MdACTIN*. Expression of the gene is presented as the fold-change in relation to its expression at 0 DAFB. Error bar represents standard error of the mean of three biological replicates Click here for file

Additional file 7 List of primer sequences used for sequencing *MdANT1* and *MdANT2*. Click here for file

Additional file 8 List of primers used for analysis of *ANTs*, *AILs*, and *MdMADS*’ gene expression with qRT-PCR. Click here for file
