# Supplementary material for: The AINTEGUMENTA genes, MdANT1 and MdANT2, are associated with the regulation of cell production during fruit growth in apple (Malus × domestica Borkh.)
Source: BMC Plant Biol. 2012 Jun 25;12:98. doi: 10.1186/1471-2229-12-98 (PMC3408378; doi:10.1186/1471-2229-12-98)
Supplement: Additional file 1 — Comparison of the predicted amino acid sequences of plant ANTs. (A) Phylogenetic analysis of two apple ANTs, and Arabidopsis ANT and AILs was performed using the neighbor joining distance method of MUSCLE. Sequences for Arabidopsis ANT and AILs were retrieved from the NCBI database. The accession numbers for Arabidopsis AILs are: AtAIL1 (AT1G72570); AtAIL2 (AT5G17430); AtAIL3 (AT3G20840); AtAIL4 (AT1G51190); AtAIL5 (AT5G57390); AtAIL6 (AT5G10510); AtAIL7 (AT5G65510) (B) Phylogenetic analysis of two ANTs and five AILs from apple. The apple AIL sequences were retrieved from the apple genome database. The accession numbers for the apple AILs are: AIL1 (MDP0000178745); AIL2 (MDP0000801540); AIL3 (MDP0000121984); AIL4 (MDP0000277643); AIL5 (MDP0000211931). (C) Phylogenetic analysis of ANTs from apple and other plants. Sequences for the ANTs used here were retrieved from the NCBI database and Genome Database for Rosaceae. Arabidopsis thaliana (AtANT; ABR21533), Brassica napus (BnANT; ABA42146), Artemisia annua (AaANT; ACY74336), Triticum aestivum (TaANT; AB458518.1), Oryza sativa (OsANT; AK106306.1), Sorghum bicolor (SbANT; XM_002468181.1), Hordeum vulgare (HvANT; AK375318.1), Malus × domestica (MdANT1), Malus × domestica (MdANT2), Prunus persica (PpANT; ppa023077m), Fragaria × ananassa (FaANT; scf0512968), Nicotiana tabacum (NtANT; AAR22388), Vitis vinifera (VvANT; AM444297), Populus trichocarpa (PtANT; AC210555). [file 1471-2229-12-98-S1.pdf]

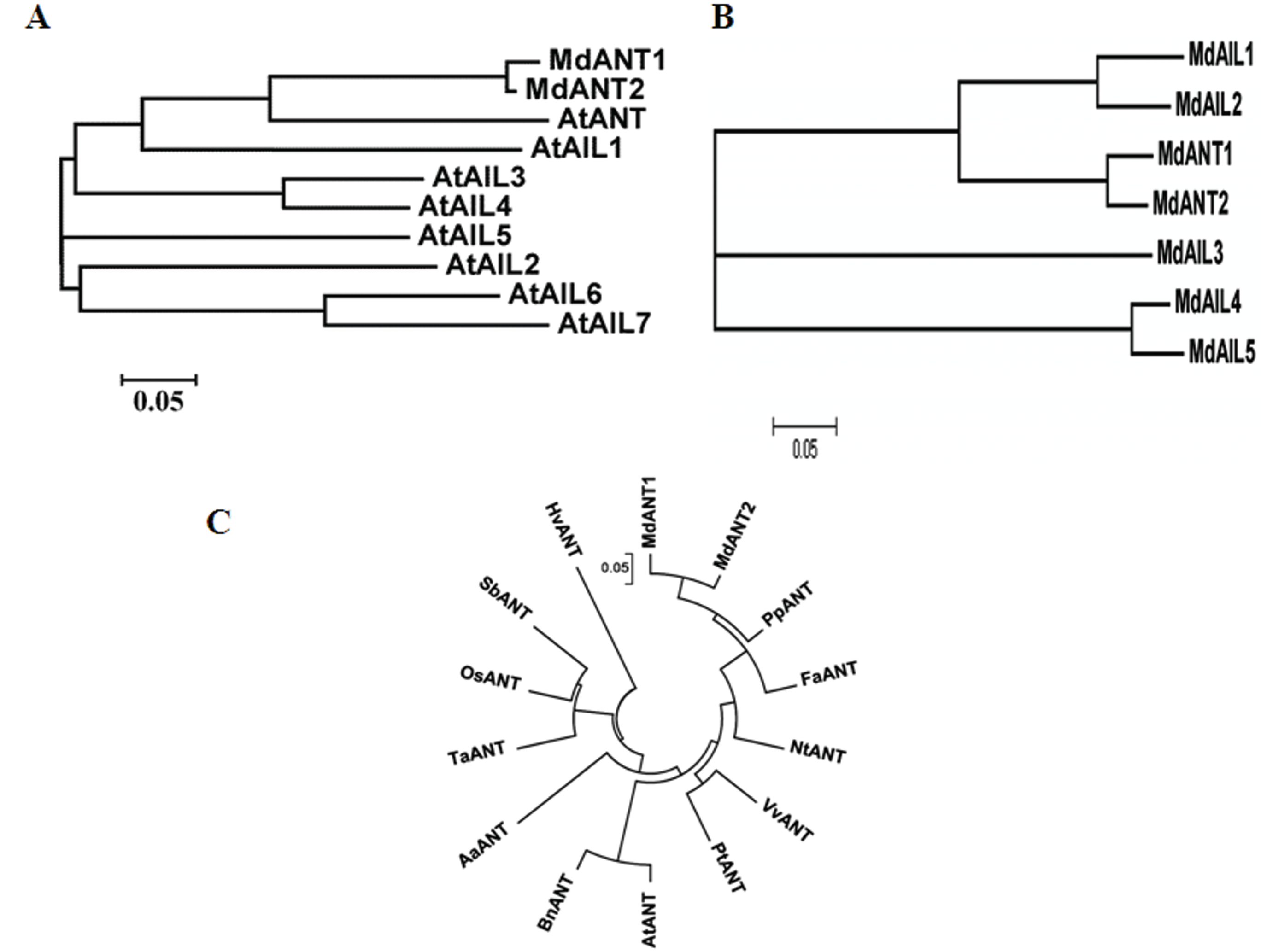


**Additional file 1: Comparison of the predicted amino acid sequences of plant ANTs.** (A) Phylogenetic analysis of two apple ANTs and Arabidopsis ANT and AILs was performed using the neighbor joining distance method of MUSCLE. Sequences for Arabidopsis ANT and AILs were retrieved from the NCBI database. The accession numbers for Arabidopsis AILs are: AtAIL1 (AT1G72570); AtAIL2 (AT5G17430); AtAIL3 (AT3G20840); AtAIL4 (AT1G51190); AtAIL5 (AT5G57390); AtAIL6 (AT5G10510); AtAIL7 (AT5G65510) (B) Phylogenetic analysis of two ANTs and five AILs from apple. The apple AIL sequences were retrieved from the apple genome database. The accession numbers for the apple AILs are: AIL1 (MDP0000178745); AIL2 (MDP0000801540); AIL3 (MDP0000121984); AIL4 (MDP0000277643); AIL5 (MDP0000211931). (C) Phylogenetic analysis of ANTs from apple and other plants. Sequences for the ANTs used here were retrieved from the NCBI database and Genome Database for Rosaceae. *Arabidopsis thaliana* (AtANT; ABR21533), *Brassica napus* (BnANT; ABA42146), *Artemisia annua* (AaANT; ACY74336), *Triticum aestivum* (TaANT; AB458518.1), *Oryza sativa* (OsANT; AK106306.1), *Sorghum bicolor* (SbANT; XM_002468181.1), *Hordeum vulgare* (HvANT; AK375318.1), *Malus × domestica* (MdANT1), *Malus × domestica* (MdANT2), *Prunus persica* (PpANT; ppa023077m), *Fragaria × ananassa* (FaANT; scf0512968), *Nicotiana tabacum* (NtANT; AAR22388), *Vitis vinifera* (VvANT; AM444297), *Populus trichocarpa* (PtANT; AC210555).
