# Supplementary material for: The AINTEGUMENTA genes, MdANT1 and MdANT2, are associated with the regulation of cell production during fruit growth in apple (Malus × domestica Borkh.)
Source: BMC Plant Biol. 2012 Jun 25;12:98. doi: 10.1186/1471-2229-12-98 (PMC3408378; doi:10.1186/1471-2229-12-98)
Supplement: Additional file 2 — Growth and gene expression during fruit development in ‘Gala’. The table displays data corresponding to Figure 2 for Fruit diameter (Figure 2A), Cell layers (Figure 2B), Relative cell production rate (RCPR; Figure 2C) and Cell area (Figure 2D). The table also displays expression data for MdANT1 and MdANT2 from Figure 3. Fruit diameter was not measured at 10 days after full bloom (DAFB). RCPR data were rounded off to the third decimal point. Gene expression was normalized using MdGAPDH and MdACTIN. Expression of a gene relative to its expression at 0 DAFB is presented. The mean and standard error of four biological replicates are displayed. [file 1471-2229-12-98-S2.pdf]

Additional File 2: Growth and gene expression during fruit development in 'Gala'. The table displays data corresponding to Figure 2 for Fruit diameter (Figure 2A), Cell layers (Figure 2B), Relative cell production rate (RCPR; Figure 2C) and Cell area (Figure 2D). The table also displays expression data for *MdANT1* and *MdANT2* from Figure 3. Fruit diameter was not measured at 10 days after full bloom (DAFB). RCPR data were rounded off to the third decimal point. Gene expression was normalized using *MdGAPDH* and *MdACTIN*. Expression of a gene relative to its expression at 0 DAFB is presented. The mean and standard error of four biological replicates are displayed.

DAFB	Fruit diameter (mm)	Cell layers	RCPR (cell cell^{-1} day^{-1})	Cell area (× 1000 μm^2)	*MdANT1* (Relative expression)	*MdANT2* (Relative expression)
0	2.7 ± 0.03	13.7 ± 0.51		0.22 ± 0.009	1 ± 0.07	1 ± 0.03
7	3.5 ± 0.05	15.3 ± 0.53	0.02 ± 0.003	0.23 ± 0.004	1.39 ± 0.17	1.36 ± 0.26
10	-	27.97 ± 0.80	0. 20 ± 0.003	0.27 ± 0.01	0.93 ± 0.17	0.92 ± 0.05
15	5.7 ± 0.16	55.1 ± 0.69	0.14 ± 0.006	0.39 ± 0.006	1.12 ± 0.12	0.97 ± 0.17
25	15.5 ± 0.32	68.8 ± 1.02	0.02 ± 0.005	0.73 ± 0.006	0.15 ± 0.05	0.38 ± 0.07
32	21.3 ± 0.22	76.8 ± 2.14	0.02 ± 0.002	1.60 ± 0.06	0.47 ± 0.03	0.38 ± 0.03
39	26.3 ± 0.22	78.1 ± 1.66	0.002 ± 0.001	3.00 ± 0.07	0.10 ± 0.03	0.20 ± 0.06
57	33.6 ± 0.34	80 ± 0.52	0.001 ± 0.001	5.67 ± 0.11	0.11 ± 0.02	0.21 ± 0.02
86	54.9 ± 0.94	79.8 ± 0.22	0.000 ± 0.000	17.31 ± 0.52	0.03 ± 0.01	0.09 ± 0.02
123	67.3 ± 1.74	80.3 ± 0.66	0.000 ± 0.000	20.67 ± 0.56	0.01 ± 0.004	0.004 ± 0.001
