# Supplementary material for: The AINTEGUMENTA genes, MdANT1 and MdANT2, are associated with the regulation of cell production during fruit growth in apple (Malus × domestica Borkh.)
Source: BMC Plant Biol. 2012 Jun 25;12:98. doi: 10.1186/1471-2229-12-98 (PMC3408378; doi:10.1186/1471-2229-12-98)
Supplement: Additional file 3 — Expression of the AIL genes during fruit development in ‘Gala’. The normalization factor was determined as the geometric mean of expression of MdGAPDH and MdACTIN. Fold change in expression is presented relative to expression during full bloom. Error bar represents the standard error of the mean of four biological replicates (n = 4). [file 1471-2229-12-98-S3.pdf]

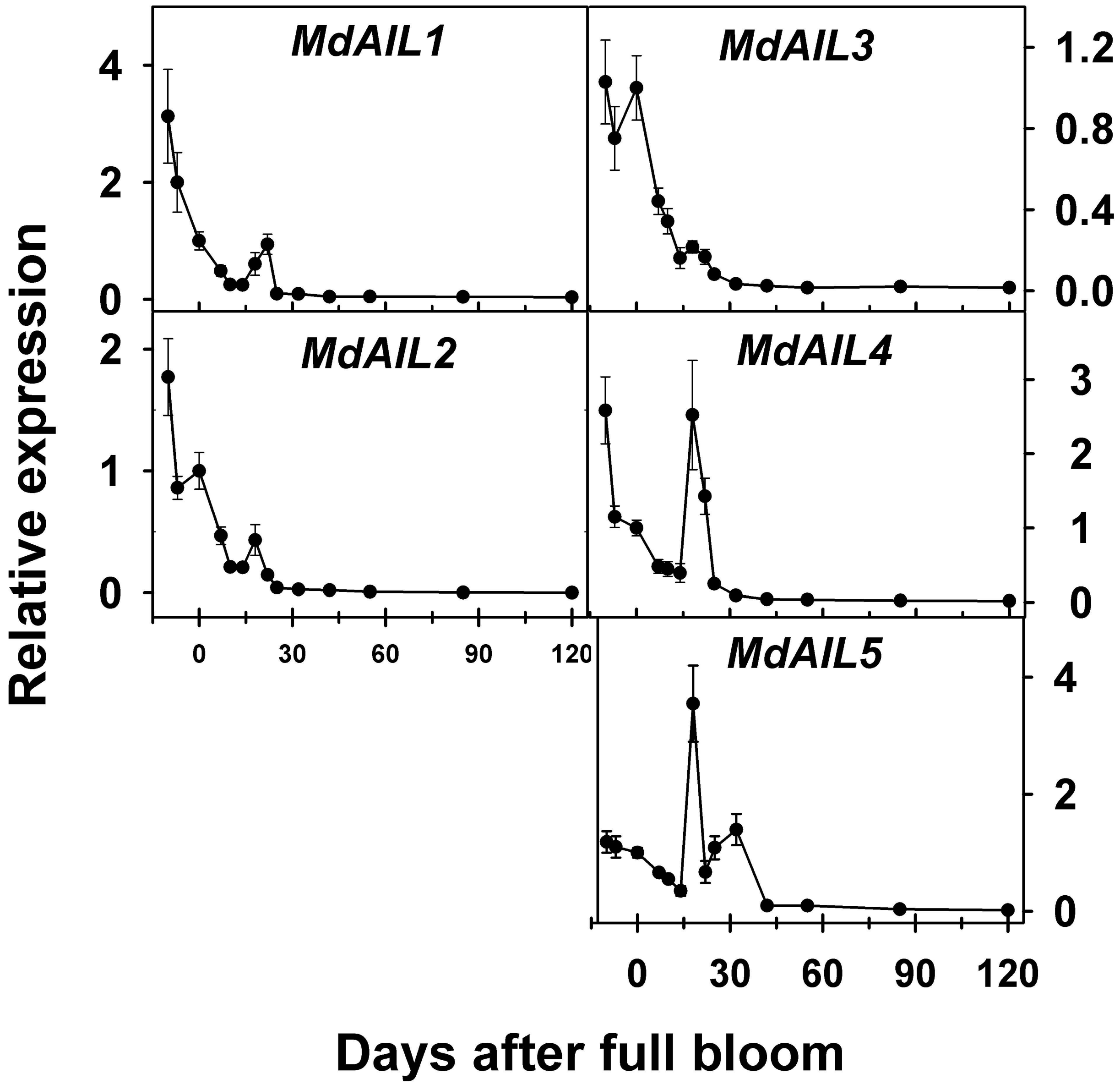

Additional file 3: Expression of the *AIL* genes during fruit development in 'Gala'. The normalization factor was determined as the geometric mean of expression of *MdGAPDH* and *MdACTIN*. Fold change in expression is presented relative to expression during full bloom. Error bar represents the standard error of the mean of four biological replicates (*n*=4).
