# Supplementary material for: The AINTEGUMENTA genes, MdANT1 and MdANT2, are associated with the regulation of cell production during fruit growth in apple (Malus × domestica Borkh.)
Source: BMC Plant Biol. 2012 Jun 25;12:98. doi: 10.1186/1471-2229-12-98 (PMC3408378; doi:10.1186/1471-2229-12-98)
Supplement: Additional file 4 — Growth and gene expression in thinned [A] and un-thinned fruit [B] of ‘Golden Delicious Smoothee’. The table shows data corresponding to Figure 4 for Fruit diameter (Figure 4A), Cell layers (Figure 4B), Relative cell production rate (RCPR; Figure 4C) and Cell area (Figure 4D). The table also shows expression data for MdANT1 and MdANT2 from Figure 5. Fruit diameter was not measured at 11 days after full bloom (DAFB). RCPR data were rounded off to the third decimal point. Expression of a gene is presented relative to its expression at 0 DAFB in thinned fruit. Gene expression was normalized using MdGAPDH and MdACTIN. The mean and standard error of four biological replicates are presented. [file 1471-2229-12-98-S4.pdf]

Additional File 4: Growth and gene expression in thinned [A] and un-thinned fruit [B] of 'Golden Delicious Smoothee'. The table shows data corresponding to Figure 4 for Fruit diameter (Figure 4A), Cell layers (Figure 4B), Relative cell production rate (RCPR; Figure 4C) and Cell area (Figure 4D). The table also shows expression data for *MdANT1* and *MdANT2* from Figure 5. Fruit diameter was not measured at 10 days after full bloom (DAFB). RCPR data were rounded off to the third decimal point. Expression of a gene is presented relative to its expression at 0 DAFB in thinned fruit. Gene expression was normalized using *MdGAPDH* and *MdACTIN*. The mean and standard error of four biological replicates are presented.

A.

DAFB	Fruit diameter (mm)	Cell layers	RCPR (cell cell^{-1} day^{-1})	Cell area (× 1000 μm^2)	*MdANT1* (Relative expression)	*MdANT2* (Relative expression)
0	3.2 ± 0.03	12.7 ± 0.73		0.30 ± 0.01	1.00 ± 0.21	1.00 ± 0.13
8	5.5 ± 0.03	23.7 ± 0.60	0.09 ± 0.006	0.38 ± 0.002	1.17 ± 0.22	1.67 ± 0.39
11	-	42.7 ± 0.60	0.20 ± 0.013	0.48 ± 0.05	0.91 ± 0.16	0.85 ± 0.10
18	11.9 ± 0.27	76.3 ± 0.89	0.09 ± 0.003	0.58 ± 0.01	0.71 ± 0.18	1.22 ± 0.14
25	20.2 ± 0.39	120.8 ± 1.07	0.06 ± 0.003	1.47 ± 0.04	1.04 ± 0.2	2.92 ± 0.62
32	26.5 ± 0.29	122.4 ± 1.05	0.002 ± .001	3.16 ± 0.11	0.29 ± 0.08	0.69 ± 0.08
50	41.0 ± 0.4	124.1 ± 0.34	0.001 ± 0.001	5.16 ± 0.17	0.12 ± 0.02	0.30 ± 0.05
79	59.9 ± 0.19	122.5 ± 0.28	0.000 ± 0.000	18.13 ± 0.74	0.03 ± 0.007	0.06 ± 0.02
128	78.7 ± 0.55	125.0 ± 1.73	0.000 ± 0.000	24.89 ± 0.46	0.21 ± 0.05	0.06 ± 0.02
150	83.0 ± 0.25	123.5 ± 0.34	0.000 ± 0.000	45.08 ± 2.33	0.01 ± 0.002	0.02 ± 0.003

B.

DAFB	Fruit diameter (mm)	Cell layers	RCPR (cell $cell^{-1}$ day^{-1})	Cell area (× 1000 μm^2)	*MdANT1* (Relative expression)	*MdANT2* (Relative expression)
0	3.1 ± 0.04	12.8 ± 0.19		0.32 ± 0.01	0.97 ± 0.16	0.76 ± 0.09
8	5.6 ± 0.18	23.4 ± 0.44	0.09 ± 0.003	0.38 ± 0.01	1.11 ± 0.25	1.62 ± 0.5
11	-	41.6 ± 0.50	0.19 ± 0.006	0.46 ± 0.01	1.06 ± 0.18	1.15 ± 0.21
18	11.1 ± 0.78	73.5 ± 0.69	0.07 ± 0.006	0.58 ± 0.01	0.80 ±0.16	1.12 ± 0.17
25	17.4 ± 0.98	86.0 ± 1.19	0.01 ± 0.004	1.4 ± 0.03	0.55 ± 0.16	0.62 ± 0.09
32	23.5 ± 0.86	90.3 ± 0.49	0.004 ± 0.003	3.7 ± 0.40	0.10 ± 0.02	0.38 ± 0.06
50	36.5 ± 0.32	92.1 ± 0.24	0.001 ± 0.001	5.8 ± 0.11	0.13 ± 0.02	0.16 ± 0.03
79	50.6 ± 1.16	92.0 ± 0.41	0.000 ± 0.000	19.9 ± 1.24	0.02 ± 0.01	0.05 ± 0.01
128	67.1 ± 1.05	92.1 ± 0.14	0.000 ± 0.000	22.3 ± 0.54	0.16 ± 0.03	0.05 ± 0.01
150	71.8 ± 1.41	93.2 ± 0.3	0.000 ± 0.000	40.8 ± 1.82	0.01 ±0.002	0.01 ± 0.003
