# Supplementary material for: The AINTEGUMENTA genes, MdANT1 and MdANT2, are associated with the regulation of cell production during fruit growth in apple (Malus × domestica Borkh.)
Source: BMC Plant Biol. 2012 Jun 25;12:98. doi: 10.1186/1471-2229-12-98 (PMC3408378; doi:10.1186/1471-2229-12-98)
Supplement: Additional file 5 — Fruit growth and gene expression in ‘Gala’ [A] and ‘Golden Delicious Smoothee’ [B]. The table displays data corresponding to Figure 6 for Fruit diameter (Figure 6A), Cell layers (Figure 6B), Relative cell production rate (RCPR; Figure 6C) and Cell area (Figure 6D). Fruit diameter was not measured at 19 growing degree days (GDD) after full bloom in ‘Gala’ and at 73 GDD after full bloom in ‘Golden Delicious Smoothee’. RCPR data was rounded off to the third decimal point. The table also shows expression data for MdANT1 and MdANT2 from Figure 7. Expression of a gene is presented relative to its expression at 0 GDD in ‘Gala’. Gene expression was normalized using MdGAPDH and MdACTIN. Data for ‘Golden Delicious Smoothee’ are from thinned fruit only. The mean and standard error of four biological replicates are presented here. [file 1471-2229-12-98-S5.pdf]

Additional File 5: Fruit growth and gene expression in 'Gala' [A] and 'Golden Delicious Smoothee' [B]. The table displays data corresponding to Figure 6 for Fruit diameter (Figure 6A), Cell layers (Figure 6B), Relative cell production rate (RCPR; Figure 6C) and Cell area (Figure 6D). Fruit diameter was not measured at 19 growing degree days (GDD) after full bloom in 'Gala' and at 73 GDD after full bloom in 'Golden Delicious Smoothee'. RCPR data was rounded off to the third decimal point. The table also shows expression data for *MdANT1* and *MdANT2* from Figure 7. Expression of a gene is presented relative to its expression at 0 GDD in 'Gala'. Gene expression was normalized using *MdGAPDH* and *MdACTIN*. Data for 'Golden Delicious Smoothee' are from thinned fruit only. The mean and standard error of four biological replicates are presented here.

A.

GDD	Fruit diameter (mm)	Cell layers	RCPR (cell cell^{-1} degree day^{-1})	Cell area (× 1000 μm^2)	*MdANT1* (Relative expression)	*MdANT2* (Relative expression)
0	2.7 ± 0.03	13.7 ± 0.51		0.22 ± 0.009	1 ± 0.07	1 ± 0.03
15.0	3.5 ± 0.05	15.3 ± 0.53	0.008 ± 0.003	0.23 ± 0.004	1.39 ± 0.17	1.36 ± 0.26
19.3	-	27.97 ± 0.80	0.14 ± 0.01	0.27 ± 0.01	0.93 ± 0.17	0.92 ± 0.05
62.6	5.7 ± 0.16	55.1 ± 0.69	0.016 ± 0.000	0.39 ± 0.006	1.12 ± 0.12	0.97 ± 0.17
143.6	15.5 ± 0.32	68.8 ± 1.02	0.003 ± 0.000	0.73 ± 0.006	0.15 ± 0.05	0.38 ± 0.07
198.1	21.3 ± 0.22	76.8 ± 2.14	0.002 ± 0.000	1.60 ± 0.06	0.47 ± 0.03	0.38 ± 0.03
237.7	26.3 ± 0.22	78.1 ±	0.000 ±	3.00 ± 0.07	0.10 ± 0.03	0.20 ± 0.06

		1.66	0.001			
417.8	33.6 ± 0.34	80 ± 0.52	0.000 ± 0.000	5.67 ± 0.11	0.11 ± 0.02	0.21 ± 0.02
759.6	54.9 ± 0.94	79.8 ± 0.22	0.000 ± 0.000	17.31 ± 0.52	0.03 ± 0.01	0.09 ± 0.02
1187.2	67.3 ± 1.74	80.3 ± 0.66	0.000 ± 0.000	20.67 ± 0.56	0.01 ± 0.004	0.004 ± 0.001

B.

GDD	Fruit diameter (mm)	Cell layers	RCPR (cell cell^{-1} degree day^{-1})	Cell area (× 1000 μm^2)	*MdANT1* (Relative expression)	*MdANT2* (Relative expression)
0	3.2 ± 0.03	12.7 ± 0.73		0.30 ± 0.01	0.94 ± 0.14	1.32 ± 0.13
48.8	5.5 ± 0.03	23.7 ± 0.60	0.013 ± 0.001	0.38 ± 0.002	1.20 ± 0.21	1.73 ± 0.14
73.3	-	42.7 ± 0.60	0.024 ± 0.002	0.48 ± 0.05	1.19 ± 0.17	0.94 ± 0.08
129.9	11.9 ± 0.27	76.3 ± 0.89	0.010 ± 0.000	0.58 ± 0.01	0.93 ± 0.23	1.35 ± 0.12
184.4	20.2 ± 0.39	120.8 ± 1.07	0.008 ± 0.000	1.47 ± 0.04	1.36 ± 0.23	3.23 ± 0.52
223.9	26.5 ± 0.29	122.4 ± 1.05	0.000 ±	3.16 ± 0.11	0.38 ± 0.1	0.77 ± 0.07

			0.000			
404.0	41.0 ± 0.40	124.1 ± 0.34	0.000 ± 0.000	5.16 ± 0.17	0.16 ± 0.03	0.33 ± 0.04
745.8	59.9 ± 0.19	122.5 ± 0.28	0.000 ± 0.000	18.13 ± 0.74	0.04 ± 0.01	0.07 ± 0.01
1316.2	78.7 ± 0.55	125.0 ± 1.73	0.000 ± 0.000	24.89 ± 0.46	0.46 ± 0.11	0.06 ± 0.01
1544.0	83.0 ± 0.25	123.5 ± 0.34	0.000 ± 0.000	45.08 ± 2.33	0.02 ± 0.01	0.02 ± 0.004
