# Supplementary material for: The AINTEGUMENTA genes, MdANT1 and MdANT2, are associated with the regulation of cell production during fruit growth in apple (Malus × domestica Borkh.)
Source: BMC Plant Biol. 2012 Jun 25;12:98. doi: 10.1186/1471-2229-12-98 (PMC3408378; doi:10.1186/1471-2229-12-98)
Supplement: Additional file 6 — Expression of MdMADS5 and MdMADS10 in the core and the cortex tissues of ‘Gala’ apple during flower and early fruit development. Closed circles represent cortex tissues and open circles represent core tissues. Core and cortex tissues were separated using laser capture microdissection. Gene expression was determined using quantitative RT-PCR and was normalized using MdACTIN. Expression of the gene is presented as the fold-change in relation to its expression at 0 DAFB. Error bar represents standard error of the mean of three biological replicates [file 1471-2229-12-98-S6.pdf]

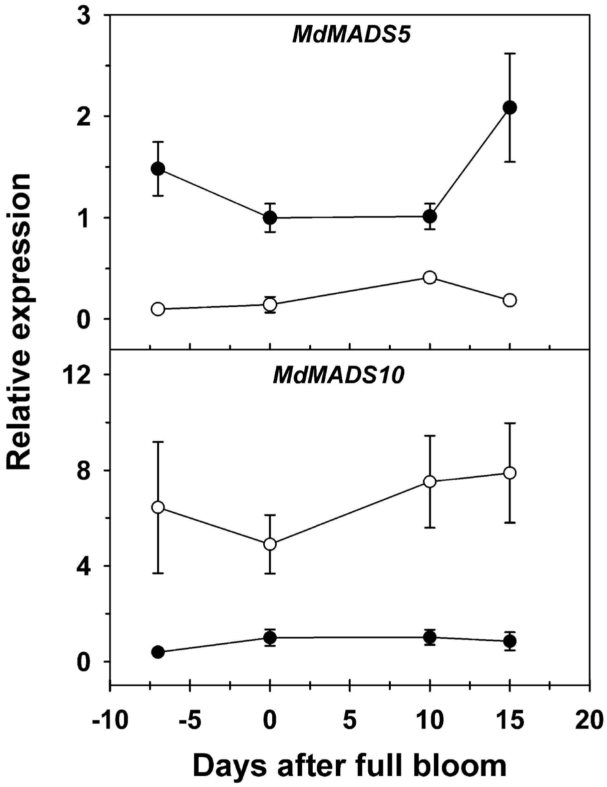

Additional file 6: Expression of *MdMADS5* and *MdMADS10* in the core and the cortex tissues of 'Gala' apple during flower and early fruit development. Closed circles represent cortex tissues and open circles represent core tissues. Core and cortex tissues were separated using laser capture microdissection. Gene expression was determined using quantitative RT-PCR and was normalized using *MdACTIN*. Expression of the gene is presented as the fold-change in relation to its expression at 0 DAFB. Error bar represents standard error of the mean of three biological replicates.
