# Supplementary material for: The AINTEGUMENTA genes, MdANT1 and MdANT2, are associated with the regulation of cell production during fruit growth in apple (Malus × domestica Borkh.)
Source: BMC Plant Biol. 2012 Jun 25;12:98. doi: 10.1186/1471-2229-12-98 (PMC3408378; doi:10.1186/1471-2229-12-98)
Supplement: Additional file 7 — List of primer sequences used for sequencing MdANT1 and MdANT2. [file 1471-2229-12-98-S7.pdf]

Additional file 7: List of primer sequences used for sequencing *MdANT1* and *MdANT2*.

Primer Name and Orientation	Type of Primer	Primer Sequence 5′-3′
NANT Reverse	Gene specific primer for PCR amplification of *MdANT1*5′ end	CCAATGCCGTTGAGAAGGAAGGG
NANT' Reverse	Gene specific primer for PCR amplification of *MdANT2* end	TCCTCCAATGCCATTGAGAATGAGAGA
DP1 Forward	Degenerate primer for 5′ sequencing	ATGCCRCTNARRTCNGAYGG
DP2 Forward	Degenerate primer for 5′ sequencing	CCNAARCTNGARGAYTTYTT
GSANT1 Reverse	Gene specific primer for PCR amplification of *MdANT1*	GGGTTATGCTCAATGGCCAGG
GSANT2 Reverse	Gene specific primer for PCR amplification of *MdANT2*	TTATACTCAATGGCTGGCGCTG
PANTF1 Forward	Primer designed from Peach *ANT*	GTTCTCACTCTCACCCCACATGAA
PANTF2 Forward	Primer designed from Peach *ANT*	GTTGCTTCTGACCCTCATCAGCAT

PANTF3 Forward	Primer designed from Peach *ANT*	GGTCAGGCTTCTTCAGCTGCTG
PANTF4 Forward	Primer designed from Peach *ANT*	ATGAATGATCACAATAATAACAACAATGGA
PANTF5 Forward	Primer designed from Peach *ANT*	AACTGGTTGGGGTTCTCACTCTC
PANTR1 Reverse	Primer designed from Peach *ANT*	GTCACGCCTCGGTACATTGAAGC
PANTR2 Reverse	Primer designed from Peach *ANT*	CCAAGATAAAGATCCTTGTTCCCAGC
PANTR3 Reverse	Primer designed from Peach *ANT*	TCAAAGTTGGTGACCGCATTTGCG
S1Primer Forward	Forward primer for full length sequencing of *MdANT1*	CTGTCTTTAGAGAGAGAAACACAGTG
S2Primer Forward	Forward primer for full length sequencing of *MdANT2*	TGTGAGTGCATAGAAGGAAGTGTAT
S2PrimerR1 Reverse	Reverse primer for full length sequencing of *MdANT2*	CTCCACTAATTACTTAACCCTCACCTC
S2PrimerR2 Reverse	Reverse primer for full length sequencing of	CATGCAAAAATCTTTGAAGGCATTTCAG

	MdANT2	
S1PrimerR1 Reverse	Reverse primer for full length sequencing of *MdANT1*	AGAATTTCCTCCACTAATTACTTACCCTAA
S1PrimerR2 Reverse	Reverse primer for full length sequencing of *MdANT1*	AATTTCTTCCCATTTTTCCTTGTTCAAT
