# Supplementary material for: The AINTEGUMENTA genes, MdANT1 and MdANT2, are associated with the regulation of cell production during fruit growth in apple (Malus × domestica Borkh.)
Source: BMC Plant Biol. 2012 Jun 25;12:98. doi: 10.1186/1471-2229-12-98 (PMC3408378; doi:10.1186/1471-2229-12-98)
Supplement: Additional file 8 — List of primers used for analysis of ANTs, AILs, and MdMADS’ gene expression with qRT-PCR. [file 1471-2229-12-98-S8.pdf]

Additional file 8: List of primers used for analysis of *ANTs*, *AILs*, and *MdMADS*' gene expression with qRT-PCR.

Gene	Primer Orientation	Primer Sequence (5′-3′)
MdAIL1	Forward	CCTCAGAACCCATGCGATGATCTTG
	Reverse	GCCATGTTGTTCTGGTCCATGGAA
MdAIL2	Forward	CATAACACGGTATGATGTGGACCGA
	Reverse	TCCCCATTGCCGATTTGCGAA
MdAIL3	Forward	CTTCGGCCAACGCACATCCATTTA
	Reverse	AGATCGTAGGCTCTTGCTGCCTTT
MdAIL4	Forward	GCGGCCATAAAGTTTAGGGGCATT
	Reverse	TCTGCTCTTCAGCTTCGAGTGAGAG
MdAIL5	Forward	CTCCCTTCTTGTCTGCACCACTTC
	Reverse	TCTTCGGGCTGAAATAAAGCGAAACTTG
MdANT1	Forward	CACCAAGGTGATCGAACCTAACATCCTG
	Reverse	CCAATGCCGTTGAGAAGGAAGGG
MdANT2	Forward	CCAAGGTGATCGAACCTAACATTGCAG
	Reverse	TCCTCCAATGCCATTGAGAATGAGAGA
MdMADS5	Forward	ATCCATCTCTGAGCTTCAGAGAAAGAG
	Reverse	GCTGTGGAAGCAGGTCAAGGC
MdMADS10	Forward	CACTTAATGGGAGATGCCTTGAGCACT
	Reverse	GCCTCTCGACTTCTGATACCTTAGTTCG
